# Iran’s Contribution to Human Proteomic Research

**DOI:** 10.22074/cellj.2019.6303

**Published:** 2019-06-15

**Authors:** Anna Meyfour, Mahya Hosseini, Hamid Sobhanian, Sara Pahlavan

**Affiliations:** 1 Basic and Molecular Epidemiology of Gastrointestinal Disorders Research Center, Research Institute for Gastroenterology and Liver Diseases, Shahid Beheshti University of Medical Sciences, Tehran, Iran; 2 Department of Molecular Systems Biology, Cell Science Research Center, Royan Institute for Stem Cell Biology and Technology, ACECR, Tehran, Iran; 3 Department of Stem Cells and Developmental Biology, Cell Science Research Center, Royan Institute for Stem Cell Biology and Technology, ACECR, Tehran, Iran; 4 Department of Biology, Payame Noor University, Tehran, Iran

**Keywords:** Infertility, Iran, Proteomics, Stem Cell

## Abstract

Proteomics is a powerful approach to study the whole set of proteins expressed in an organism, organ, tissue or cell resulting in valuable information on physiological or pathological state of a biological system. High throughput proteomic data facilitated the understanding of various biological systems with respect to normal and pathological conditions particularly in the instances of human clinical manifestations. The important role of proteins as the functional gene products encouraged scientists to apply this technology to gain a better understanding of extremely complex biological systems. In last two decades, several proteomics teams have been gradually formed in Iran. In this review, we highlight the most important findings of proteomic research groups in Iran at various areas of stem cells, Y chromosome, infertility, infectious disease and biomarker discovery.

## Introduction

Proteomics is an important field of biological research that studies the whole set of proteins expressed in an organism, organ, tissue or cell resulting in valuable information on physiological or pathological state of a biological system. Thus, it can help the health system via understanding the pathogenesis of diseases (1), discovery of novel biomarkers (2), identification of therapeutic target candidates (3, 4) and drug discovery (5, 6). The application of proteomics in various field of biological sciences, influenced our understanding of molecular mechanisms governing living systems. Thus, many research groups applied this approach to answer multiple unsolved biological/clinical questions.

During last two decades several proteomics teams have been gradually formed in Iran and, exploited proteomics in various human research fields including stem cell science, infertility, biomarker discovery and infectious disease. Owing to significant progress in stem cell proteomics, it has been one of the most active filed of proteomics studies in Iran (7). In 2005, Iranian scientist generated the first proteome map of human embryonic stem cell membrane (hESC) (8). Since then, many studies have been performed to identify proteins involved in stem cell differentiation as well as lineage specific cell surface markers (9).

international projects. These include Human Y Chromosome Proteome Project and Asia Oceania Human Proteome Organization Initiative on hESC membrane proteome. This review highlights the most important findings of proteomic research groups in Iran at various areas of stem cells, infertility, infectious disease and biomarker discovery (Table 1).

### Proteomic research in Iran

#### Stem cell proteomics

Over last two decades, stem cell institutes of Iran have attracted major funding and recruited scientists to accelerate the development of this field, in order to improve both developmental knowledge and clinical translation for the purpose of regenerative medicine (7, 10).

hESCs are pluripotent cells that have the ability for selfrenewal and differentiation to all three embryonic germ layers. They provide an unprecedented source of cells for developmental study, disease modeling, and drug screening as well as cell-based therapies. Scientists have expressed tremendous interest in the molecular mechanisms that govern pluripotency, proliferation, and differentiation of ESCs, and employed various approaches to unravel these regulatory mechanisms (11). In Iran, proteomics has been used to study molecular mechanisms which play a role in maintaining the undifferentiated state of hESCs and induced pluripotent stem cells (iPSCs) (8, 12) as well as differentiation of monkey, mouse, and human ESC lines into embryoid bodies (13-16) and hESC neural differentiation (17, 18). The proteome of spinal cords in healthy and experimental autoimmune encephalomyelitis (EAE) samples have been analyzed before and after transplantation of ESC-derived neural precursors (19).

**Table 1:** An overview to human proteomic research in Iran


	Field	Finding	Proteomic Tool	Reference

1	Stem cell	Molecular mechanisms which play a role in maintaining the undifferentiated state of hESCs and iPSCs	2-DE-MS/MS	(8)
			2-DE-MS/MS	(12)
2		Molecular mechanisms which play a role in differentiation of mouse, monkey, and human ESC lines into embryoid bodies	2-DE-MS/MS	(13)
			2-DE-MS/MS	(14)
			iTRAQ-MS/MS	(15)
			2-DE-MS/MS	(16)
3		Molecular mechanisms which play role in neural differentiation of ESC	2D-DIGE-MS/MS	(17)
4		Molecular mechanisms of EAE recovery after transplantation of ESC-derived neural precursors	2-DE-MS/MS	(19)
5		Identification of cytosolic and membrane associated protein complexes of hESC	Blue native PAGE-MS/MS	(20)
6		Reduction of focal adhesion enables the maintenance of undifferentiated and pluripotent states of mESCs	Label-free mass spectrometry	(21)(22)
7		Identification of hundreds of organelle specific proteins and functional assignment of three novel membrane proteins to pluripotency	Label-free mass spectrometry	(23)
8		Deep subcellular proteomics resulted in identification of 15, 3, 13 gold missing proteins in membrane, cytoplasm, and nucleus of hESCs	Label-free mass spectrometry	(24)
9		Identification of ALCAM as a speciﬁc surface marker to enrich ISL1^+^ cardiac progenitor cells	Label-free mass spectrometry	(25)
10		Identification of two novel cell surface markers in order to purify Embryonic Dopamine progenitor cells	Label-free mass spectrometry	(26)
11	The Y Chromosome Human Proteome Project (Y-HPP)	Identification of two MPs (XKRY and CYORF15A) and introduction of promising molecular markers to predict retrievable sperm presence in MA patients	Western blot/Immmunohistochemistry	(27)
12		Identification of two novel splice variants of KDM5D and their possible role in prostate cancer cells’ growth	Label-free mass spectrometry	(28)
13		Identification of DDX3Y role in neural differentiation of NTERA-2	Label-free mass spectrometry	(29)
14		Identification of a missing protein, TBL1Y, and its role in cardiac differentiation of hESCs	Western blot/Immmunofluorescent	(30)
15	Infertility	Identification of proteins which are differentially expressed in spermatozoa of men with or without varicocele	2-DE-MS/MS	(31)
16		Identification of proteins which are differentially expressed in spermatozoa of patients with grade 3 varicocele before and after varicocelectomy	2-DE-MS/MS	(32)
17		Identification of 14 differentially expressed proteins in sperm tails of asthenozoospermia patients compared to normozoospermia men	2-DE-MS/MS	(33)
18		Identification of anti-sperm antibody targets in azoosprmic men	2-DE-MS/MS	(34)
19		Identification of novel candidate proteins which play role in regulation of spermatogenesis	Label-free mass spectrometry	(35)
20		Reconstruction of SpermNet	Whole-proteome data and the mCADRE algorithm	(36)
21		Identification of some proteins in endometrial tissue of PCOS patients which play important roles in fecundity and fecundability	2-DE-MS/MS	(37)
22	Infectious disease	Identification of proteins which play important roles in rabies virus pathogenesis	2-DE-MS/MS	(1, 38-40)
23		Proteomics of Leishmaniasis	2-DE-MS/MS	(41, 42)
24	Biomarker discovery	Introduction of a panel of urinary prognostic biomarkers for classification of IgA nephropathy	Label-free mass spectrometry	(43)
25		Identification of a panel of biomarkers to predict the responsiveness to steroid therapy in focal segmental glomerulosclerosis	Label-free mass spectrometry	(44)
26		Identification of a panel of biomarkers for early diagnosis as well as sensitivity to dexamethasone therapy in B cell acute lymphoblastic leukemia	2-DE-MS/MS	(45)
27		Introduction of Ig Kappa Chain C region (IGKC) as a potential biomarker for fluoxetine responsiveness and patient follow up	2-DE-MS/MS	(46)


Subcellular localization of a protein to different organelles can greatly determine the decisive protein function (47). Blue native polyacrylamide gel electrophoresis (PAGE) was used to identify cytoplasmic and membrane-associated complexes in hESCs (20).

The shotgun proteome approach was used to study ground state pluripotency. The results indicated that reduced focal adhesion enabled mouse embryonic stem cells (mESCs) to be maintained in undifferentiated and pluripotent states (21, 22).

### Asia Oceania Human Proteome Organization Initiative on hESC membrane proteome

Iranian scientists are actively involved in hESC Membrane Proteome Initiative, an Asia Oceania Human Proteome Organization (AOHUPO) initiative Chaired by Prof. Ghasem Hosseini Salekdeh from Iran and Prof. Yu Ju Chen from Taiwan.

 Although embryonic stem cells have become a cell of choice for multiple purposes from developmental studies to cell-based therapeutics, there are still unknown remaining aspects of these cells, such as a complete protein map of their membrane proteins. Researchers undertook a study on subcellular proteomics of hESC fractions using a TripleTOF mass spectrometer as part of the AOHUPO and projects related to the hESC Membrane Proteome Initiative. This study resulted in the identification of hundreds of organelle specific proteins and was followed by functional assignment of three novel membrane proteins to pluripotency (23).

 In 2018, the most comprehensive proteome map of hESC has been published as a result of this initiative. A deep subcellular proteomics approach was used to identify the membrane, cytoplasmic and nuclear proteins of hESCs in survey for identification of missing proteins (MPs). This great study revealed 15, 3, 13 gold missing MPs in membrane, cytoplasm, and nucleus fractions which determined to play role in self-renewal, regulation of differentiation, epigenetic regulations, and cellular layers development in hESCs (24).

This initiative also tuned the focus of proteomic studies toward discovering cell surface markers for the purpose of lineage-specific cell sorting. LIM-homeodomain transcription factor ISL1 is one of the main markers of cardiac progenitor cells that is believed to be the master regulator of fate determination for the secondary heart field-derived cardiac cells (48-52). To purify a population of cardiac progenitor cells, ISL1 can not be directly used due to its nuclear localization. Thus, a genetic selection strategy was used to mark ISL1+cells in order to identify a cell surface marker by label-free shotgun proteomics approach for future applications in safe clinical sorting of cardiac lineage-specific cells. ALCAM (CD166) was introduced as a cell surface marker which could successfully purify the population of ISL1+ cardiac progenitor cells with the ability to recover cardiac function and improve angiogenesis in a rat model of myocardial infarction (25).

In a similar approach, a transcription factor that marks early dopaminergic neurons, LIM homeobox transcription factor 1 alpha (LMX1A), was used to generate a knockin GFP reporter human embryonic stem cell (hESC) line in order to purify this particular neuronal population for further cell surface marker identification. Using shotgun proteomics, Fathi et. al. introduced two cell surface markers, polysialylated embryonic form of neural cell adhesion molecule (PSA-NCAM) and contactin 2 (CNTN2), which could successfully enrich LMX1A+ progenitor cells. Further transplantation of CNTN2+ cells improved Parkinson’s disease-related phenotypes in rat models (26).

### Iran’s contribution to human proteome project: the Y chromosome human proteome project

The Y chromosome human proteome project (Y-HPP) is being conducted in Iran (53). The project focuses on identification of MPs and study of the function of proteins and their association with diseases (54, 55). Rastegar and colleagues performed an isoform-level gene expression profiling of Y chromosome genes within the azoospermia factor (AZF) regions, their X counterparts, and a few autosomal paralogues in four different groups: healthy individuals with preserved spermatogenesis, patients with non-obstructive azoospermia (NOA), Sertoli-cellonly syndrome (SCOS), and premeiotic maturation arrest (MA). They identified, for the first time, two MPs (XKRY and CYORF15A). Rastegar introduced HSFY1-3, RBMX2, BPY2-1, DAZL-1, and KDM5C2 as promising molecular markers to predict retrievable sperm presence in MA patients (27). Jangravi et al. (28) studied KDM5D expression, an MSY gene, in a prostate cancer cell line (DU-145). They found two novel splice variants with lengths of 2650 bp and 2400 bp. Knockdown of these two variants resulted in higher growth and lower apoptosis rate of prostate cancer cells.

Shotgun label-free quantitative proteomics revealed alterations in abundance of proteins involved in RNA processing, protein synthesis, apoptosis, the cell cycle, growth, and proliferation in KDM5D knockdown cells. Vakilian et al. (29) investigated the expression of 23 MSY genes and 15 of their X homologues during neural cell differentiation of NTERA-2 human embryonal carcinoma cell line (NT2) cells. They observed alterations in expression of several MSY genes, from which DDX3Y was knocked down to further investigate its function during neurogenesis. Label-free quantitative shotgun proteomics showed that DDX3Y knockdown resulted in expression alterations of proteins involved in the cell cycle, RNA splicing, and apoptosis. They suggested that DDX3Y might play a multifunctional role in neural cell development. Meyfour and co-workers studied the proteome of Y chromosome during cardiogenesis of hESCs. They observed alterations in the expression and protein localization of some of the MSY genes during cardiomyocyte differentiation of hESCs from which TBL1Y, a MP, was knocked down for further functional analysis. TBL1Y knockdown resulted in inefficient cardiac differentiation of hESCs along with generation of cardiomyocytes with impaired contraction (30).

### Proteomics in infertility research

Human male infertility accounts for approximately 7-10% of infertilities (56) and is an important medical issue with an unknown etiology in most cases. Hosseinifar et al. have compared the sperm protein profiles of men with and without varicocele by Two-dimensional gel electrophoresis (2-DE) and mass spectrometry (MS). Their results indicated that heat shock, mitochondrial, and cytoskeletal proteins were mainly affected by varicocele (31). In another study, they analyzed the proteome profile from patients with varicocele and poor sperm quality before and after varicocelectomy. Their findings showed that the altered proteins played a key role in sperm production, protection of DNA integrity, and sperm motility (32). In a study by Hashemitabar et al. (33), the proteome profile of the sperm tail was analyzed in patients with normozoospermia and asthenozoospermia. For the first time, they identified four differentially expressed proteins which could be potential markers to better diagnose sperm dysfunction, new contraceptive targets, and prediction of embryo quality.

Zangbar et al. (34) profiled the sperm proteins and blotted them with sera of healthy fertile or obstructive azoospermia (OA) in order to explore the anti-sperm protein targets in azoospermic men. They observed that sera from OA patients might contain antibodies against two sperm proteins, Tektin-2 and triose phosphate isomerase. Alikhani et al. (35) performed shotgun proteomics in order to unravel the molecular mechanisms involved in male azoospermia. They compared the protein profile of testicular tissues from OA patients and NOA that included MA and SCOS. Their findings introduced novel candidate proteins, which included key transcription factors associated with azoospermia. Recently, Asghari et al. (36) reconstructed the first proteome-scale model of the sperm cell by using whole-proteome data and the Metabolic Context-specificity Assessed by Deterministic Reaction Evaluation (mCADRE) algorithm. Their model could predict the novel non-glycolytic genes for deficient energy metabolism in addition to known pathways in asthenozoospermia. Proteomics was employed to address infertility complications in female reproductive system, too. Proteome profile of endometrial tissue in polycystic ovary syndrome (PCOS) was compared to healthy fertile women and resulted in identification of 70 proteins which assigned a role in oxidative stress, inflammation, apoptosis and the cytoskeletal rearrangement that may underlie impaired fecundability and low pregnancy rate in PCOS women (37).

### Proteomics in infectious disease

Proteomics, as a novel approach, has helped the scientific community to develop a molecular picture of infectious diseases and their spread, which will definitely result in better control of disease prevalence and the development of more efficient therapies (57). Rabies is a neurodegenerative disease caused by a life threatening rabies virus. Proteomics has been applied to study the effect of the virus on baby hamster kidney cells (BHK- 21) (1), a neuroblastoma cell line (38), lymphocytes of infected mice (39), and human brain infected by the virus (40). Leishmaniasis is another infectious disease studied by proteomics (41, 42). More than 20 species of intracellular parasites that belong to the Leishmania genus cause this infectious disease. The symptoms range from simple self-limiting cutaneous ulcers to sever disfiguring mucocutaneous lesions, and even fatal visceral disease. Among these infectious diseases, the diagnosis and treatment of viscerotropic leishmaniasis appears challenging. It is anticipated that technologies developed in the course of C-HPP can be applied for research of infectious diseases in the future.

### Proteomics in biomarker discovery

Despite great advances in our understanding of epidemiology and pathophysiology of diseases, but diagnosis and therapeutic decisions for many pathological conditions rely on invasive tools. Moreover, prognosis or early detection of few disease conditions are possible by biomarker discovery. Therefore, great efforts are made to introduce ideal biomarkers to improve prognosis, diagnosis and predictive response to treatment. Advances in proteomic technologies can greatly influence the field of biomarker discovery (58). This concept has been the objective of some research projects in Iran.

The classification of immunoglobulin A (IgA) nephropathy using scoring systems showed inconsistencies among nephrologists. Kalantari et al introduced a panel of prognostic biomarkers using liquid chromatography tandem-mass spectrometry (LC-MS/ MS) which can be applied for prognosis and classification of IgA nephropathy (43). Furthermore, they found a panel of biomarkers using nano-LC-MS/MS which could help to predict the responsiveness to steroid therapy in focal segmental glomerulosclerosis (44). Dehghan-Nayeri et al. (45) identified CAPZA1, CAPZB, CLIC1, PNP and PSME1 as a panel of biomarkers for early diagnosis as well as sensitivity to dexamethasone therapy in B cell acute lymphoblastic leukemia. Zamanian Azodi et al. (46) studied proteome profile of obsessive-compulsive disorder (OCD) patients before and after treatment with fluoxetine and introduced Ig Kappa Chain C region (IGKC) as a potential biomarker for fluoxetine responsiveness and patient follow up.

### Proteomics in the mirror of global endeavor

Iranian researchers have significantly contributed to proteomic science by establishing international collaborations. They have also pioneered some research areas such as the proteome map of human embryonic stem cells. Furthermore, Iran has contributed in C-HPP as the country responsible for the proteome profile of Y chromosome and has successfully fulfilled this goal by identification of multiple Y chromosome MPs. Iranian proteomic researchers developed a new strategy for identification of MPs with highly similar homologous proteins where MS cannot provide sufficient data. Furthermore, introduction of novel surface markers for pluripotent stem cells and lineage committed cells was another important achievement of proteomic research in Iran which may facilitate the clinical translation of these cells. Moreover, recently neuroproteomics (59, 60), post translational modification (PTM) analysis (61) as well as functional characterization of identified Y chromosome proteins with no known function as ‘dark proteins’(62) gained more attention in Iran. This influential role of Iran will be continued to the future of proteomics as more international collaborations are established and higher number of motivated researchers get involved.

## Conclusion

In line with global advancement of proteomic research, Iranian scientists have been contributing to this research area by using various techniques of protein analysis. Despite limitations in high technology equipment required for proteomic studies, such as MS, this contribution continued to be in effect by establishing alternative experimental tools as well as forming international collaborations. These universal efforts have resulted in and will continue to provide valuable information directly or indirectly empowering health system by understanding the pathogenesis of diseases, discovery of novel biomarkers, identification of therapeutic target candidates and drug discovery.
